# Ventral Stabilization of a T2-T3 Vertebral Luxation via Median Sternotomy in a Dog

**DOI:** 10.1155/2018/9152394

**Published:** 2018-09-10

**Authors:** Sarah Klatzkow, Matthew D. Johnson, Michele James, Sheila Carrera-Justiz

**Affiliations:** University of Florida, College of Veterinary Medicine, Department of Small Animal Clinical Sciences, 2015 SW 16th Ave, Gainesville, FL 32610, USA

## Abstract

An 8-year-old neutered male miniature Poodle presented for evaluation of a suspected T3-L3 lesion with cervical component following vehicular trauma. Magnetic resonance imaging and computed tomography revealed a T2-T3 luxation with right displacement of T3. A T2 caudal endplate fracture was present as well as multifocal noncompressive bulges of cervical intervertebral discs. Conservative management failed and ventral stabilization of C7-T4 was performed via a median sternotomy. Paired String-of-Pearls plates were placed on the ventral aspect of vertebrae. Eight weeks postoperatively, the dog was ambulatory with moderate pelvic limb paraparesis. A luxation of T2-T3 is uncommon in small animals and surgical stabilization is poorly described in literature. This case report demonstrates the use of a ventral approach to cranial thoracic vertebral stabilization with a successful outcome.

## 1. Introduction

Spinal cord dysfunction as a result of luxation and fracture is a common sequela of trauma, with motor vehicles being the cause in 40-60% of reported cases [1–3]. These types of injuries are most commonly seen at the junction of stable and mobile regions, namely, thoracolumbar and lumbosacral [2, 4, 5]. Luxations of the C6-T2 region occur in <10% of cases while T10-L2 accounts for 50% of cases [1–3]. In conjunction with luxations, vertebral endplate fractures are seen in 22% of cases [2].

A variety of procedures have been described for stabilizing vertebral luxations and fractures in the cervical and thoracolumbar regions including dorsolateral vertebral body plating, external skeletal fixation, and a composite of polymethylmethacrylate (PMMA) with either pins or screws [1, 3–13]. The cranial thoracic region is inherently stable; injuries are uncommon and neither approach nor technique is well described.

This is, to the authors' knowledge, the first reported case of ventral vertebral body plating for stabilization of a T2-T3 spinal luxation via median sternotomy.

## 2. Case Presentation

An 8-year-old, 8.1 kg, neutered male miniature Poodle presented with suspected spinal trauma after being hit by a car 4 days prior to referral. The dog had been medically managed at a local emergency clinic with concerns of a T2-T4 injury with cervical component. Thoracic radiographs taken at the time of presentation to the emergency clinic revealed possible vertebral injury or intravertebral disc compression at T2-T3; the remaining thoracic images were unremarkable.

On initial presentation, the dog was tachypneic and laterally recumbent with a body condition score of 5/9. The dog vocalized on palpation of the neck and pain was assessed at a 2/4 using the Canine Acute Pain Scale [14]. The remainder of the examination was unremarkable.

On neurologic examination, the dog was laterally recumbent, quiet, and alert. Cranial nerve function was grossly intact. The dog was nonambulatory with paresis of the thoracic limbs and plegia of the pelvic limbs. Muscle tone was increased in the thoracic limbs. When supported in standing position, a right head turn was noted and conscious proprioception was decreased in the thoracic limbs and absent in the pelvic limbs. Segmental reflexes were intact. The panniculus reflex was absent caudal to L1 bilaterally. No pain was elicited on spinal palpation; cervical range of motion was not evaluated. Neurolocalization was determined to be T3-L3 with suspected C6-T2 involvement.

Notable blood work findings included an elevated alanine aminotransferase (185U/L; reference, 10-125U/L), normochromic normocytic anemia (hematocrit 35.7%; reference, 40-56%), leukocytosis (15.69K/uL; reference, 5-13 K/uL), neutrophilia (10.7K/uL; reference 2.7-8.9K/uL), monocytosis (2K/uL; reference, 0.1-0.8K/uL), and hyperfibrinogenemia (500mg/dL; reference, 100-400mg/dL).

Abdominal ultrasound revealed mild retroperitoneal effusion adjacent to the left kidney consistent with trauma with no other significant findings.

The dog was placed under general anesthesia for advanced imaging with propofol (2mg/kg IV) and midazolam (0.3mg/kg IV) and maintained on isoflurane. Maropitant (1mg/kg SQ) and atropine (0.01mg/kg IV) were administered as needed.

**Figure 1:**
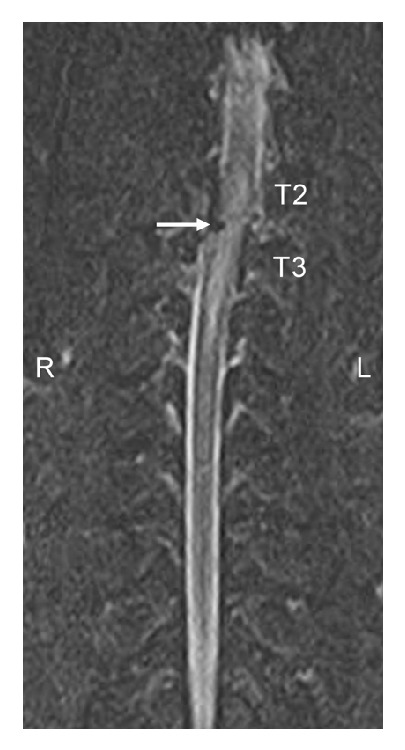
This image is a dorsal plane STIR MRI of the dog's spine revealing a T2-T3 luxation. Image reveals right lateral displacement of the caudal spine at the level of T2 and T3 vertebrae. The white arrow denotes area of suspected hemorrhage and edema within the spinal canal and spinal cord parenchyma.

Magnetic resonance imaging (MRI) of the entire spine revealed a T2-T3 vertebral luxation with focal spinal cord compression, characterized by a right lateral displacement of T3. Intramedullary spinal cord changes noted on T2 weighted image (T2-WI) and short tau inversion recovery (STIR) were suggestive of edema and/or hemorrhage, with suspected adjacent epaxial myositis. Multifocal noncompressive bulges of the C2-C7 intervertebral discs were noted (Figure 1).

**Figure 2:**
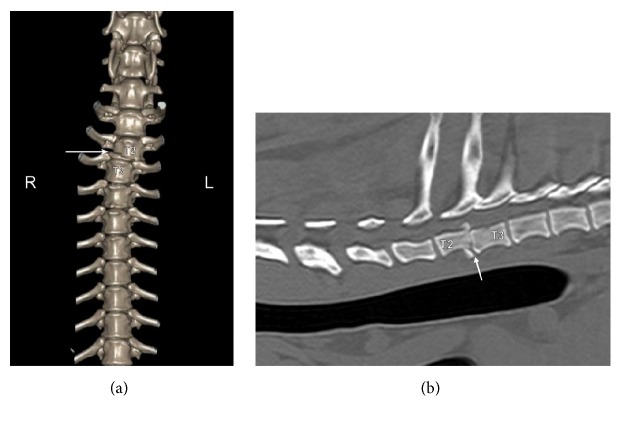
(a) Three-dimensional reconstruction of the preoperative computed tomographic imaging. Imaging of the cranial thoracic spine that shows right lateral displacement of the caudal spine at the level of T2 and T3 vertebrae. The white arrow denotes caudal end plate fracture of T2. (b) Preoperative Computer Tomographic imaging of the cranial thoracic spine at the level of the spinal subluxation. The caudal end plate fracture of T2 is noted (white arrow).

On noncontrast computed tomography (CT), a vertebral luxation of T2-T3 was confirmed, with a 4.5mm right displacement of the T3 vertebral body. A T2 caudal endplate fracture was noted with associated T2-T3 disc space collapse. A single mineralized 4mm fragment was located within the vertebral canal at the level of T2-T3 (Figure 2). The dog recovered from general anesthesia uneventfully and surgery was scheduled. Due to the severity of the spinal cord injury, the clients were counseled regarding the potential for the dog to have permanent pelvic limb deficits.

Prior to surgery, the dog was managed on methadone (0.2mg/kg IV bolus, then 0.1mg/kg IV bolus, then IV CRI at 0.025-0.05mg/kg/hr), acepromazine (0.01mg/kg IV once), and maropitant (1mg/kg IV once) and maintained at 40% O2.

**Figure 3:**
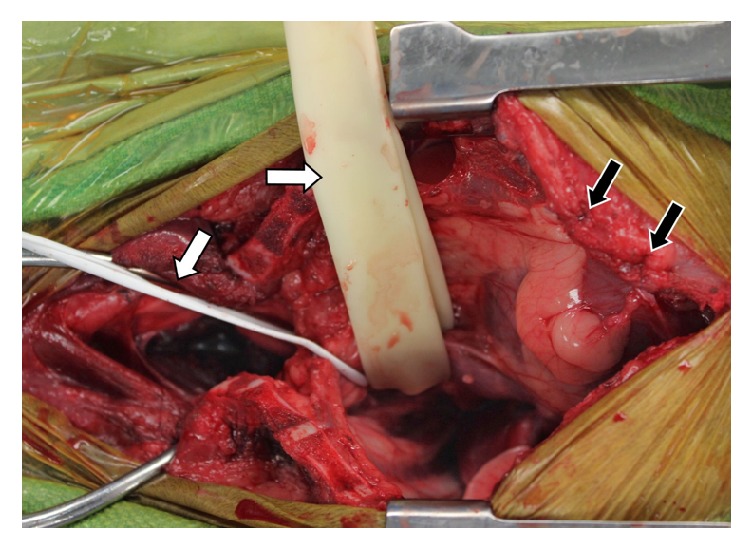
Intraoperative image. Black arrows denote sutures of the pericardial sling. White arrows denote Penrose tubing retracting vessels of the brachycephalic trunk.

**Figure 4:**
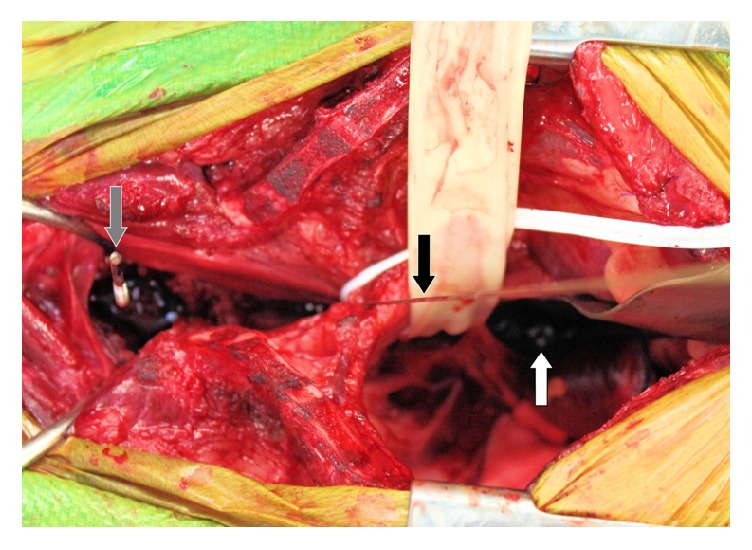
Intraoperative image. String-of-Pearls plate (white arrow) placed along ventral spine held in place with a Kirschner wire (black arrow). Grey arrow denotes a drill bit left in place to check depth with fluoroscopy.

**Figure 5:**
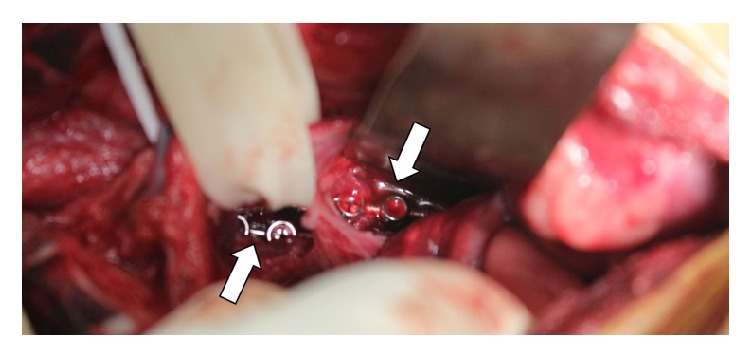
Intraoperative image showing paired, adjacent string of pearl plates (white arrows).

**Figure 6:**
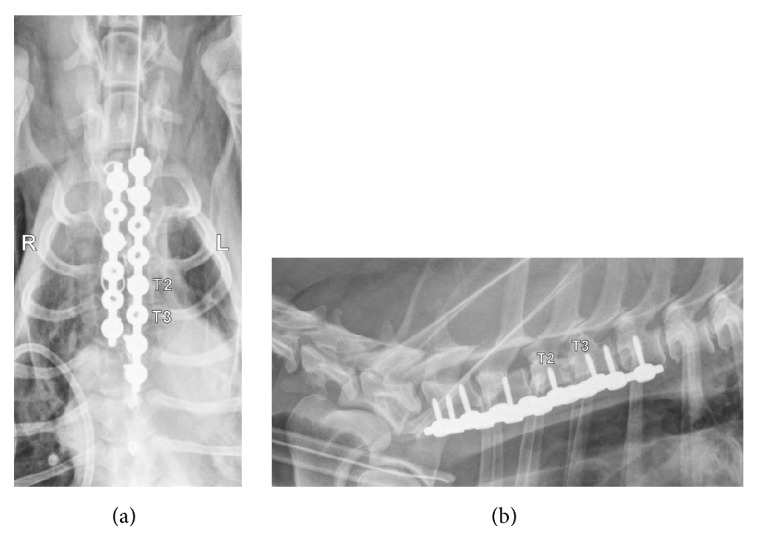
(a) Postoperative ventrodorsal radiographic projection of the cranial thoracic spine at the level of the spinal subluxation. Juxtaposed string of pearl plates are secured from the C7 to T4 vertebral bodies. The 8-hole plate on the dog's left engages vertebral bodies C7, T2, T3, and T4. The 6-hole plate on the dog's right side engages vertebral bodies C7, T1, and T3. Two wires are noted from the medial sternotomy closure superimposed over the 6-hole plate. (b) Postoperative lateral radiographic projection of the cranial thoracic spine at the level of the spinal subluxation. Screws are noted to engage vertebral bodies of C7-T2 cranial to the injury. Three screws engage vertebrae T3 and T4.

It was elected to stabilize C7-T4 and the dog was premedicated with methadone (0.3mg/kg IV) and lidocaine (2.5mg/kg IV) and induced with propofol (3mg/kg IV) and ketamine (3mg/kg IV). The dog was maintained on isoflurane and received atropine (0.4mg/kg IV), a combination of hydromorphone, lidocaine, and ketamine (end total 1.32mg, 131mg, and 44mg IV CRI, respectively), dopamine (total 2mg IV CRI), cefazolin (22mg/kg q90min), and packed red blood cells (100mL IV). A ventral cervical approach was extended caudally into a median sternotomy to gain adequate exposure. The mediastinum and brachycephalic trunk were dissected bluntly to approach the ventral spinal musculature. A pericardial sling was employed to allow caudal and ventral retraction of the heart and great vessels away from the area of interest. Similarly, retractors were placed near the thoracic inlet and caudally along the brachycephalic trunk to increase visualization of the spine and prevent vital structures from becoming entrapped with the various power instruments utilized (Figure 3). The longus coli muscles were elevated from the ventral aspect of C6-T5. Manual reduction of the luxation was attempted; however due to the presence of fibrous tissue, complete reduction of the lesion was not achieved. An 8-hole 2.0mm String-of-Pearls (SOP) plate (OrthoMed, West Yorkshire, UK) was placed along the ventral aspect of C7-T4 vertebral bodies and temporarily stabilized with 0.89mm Kirschner wires (IMEX Veterinary, Longview, Texas) placed through a node on both sides of the T2-T3 disc space (Figure 4). Plate location and pin depth were evaluated with fluoroscopy (Insight II; Hologic, Marlborough, Massachusetts). A 6-hole 2.0mm SOP plate was contoured to the vertebral surfaces of C7-T3 and placed adjacent to the first plate. 2.0mm cortical screws (DePuy Synthes, West Chester, Pennsylvania) were placed in holes engaging C7, T1, and T3 in the 6-hole plate (Figure 4). Cortical screws were placed in the 8-hole plate engaging C7 and T2-T4 following removal of the Kirschner wires (Figure 5). Based on the String-of-Pearls plate design as a locking construct, the screws were placed with the intention of being monocortical; however second cortex was breached in the T3 and T4 vertebrae. A 5 Fr MILA chest tube (International, Erlanger, Kentucky) and subcutaneous soaker catheter (MILA International, Erlanger, Kentucky) were placed and closure was routine. Postoperative radiographs indicated apparent appropriate implant placement, though the T2-T3 disc space remained collapsed (Figure 6).

The dog was managed postoperatively with fentanyl (1-5mcg/kg/h IV CRI), naloxone (0.04mg/kg IV once), bupivacaine (1mg/kg q8h through soaker catheter), dexamethasone SP (0.2mg/kg IV once), prednisone (0.6mg/kg PO q24h), and cefazolin (22mg/kg IV q8h). The day following surgery, tramadol (3mg/kg PO q8h) was initiated; fentanyl and cefazolin were discontinued. The dog's neurologic status remained unchanged immediately postoperatively. Three days later, the dog was transferred to his primary veterinarian for long-term care.

The dog re-presented eight weeks after surgery for follow-up. Physical examination was unremarkable. Neurologic examination revealed an ambulatory patient with moderate to marked pelvic limb paraparesis and proprioceptive ataxia. Conscious proprioception was absent in pelvic limbs bilaterally. Segmental reflexes and panniculus were intact. No pain was elicited on spinal palpation. The lesion was neurolocalized to T3-L3 and markedly improved from prior exam.

**Figure 7:**
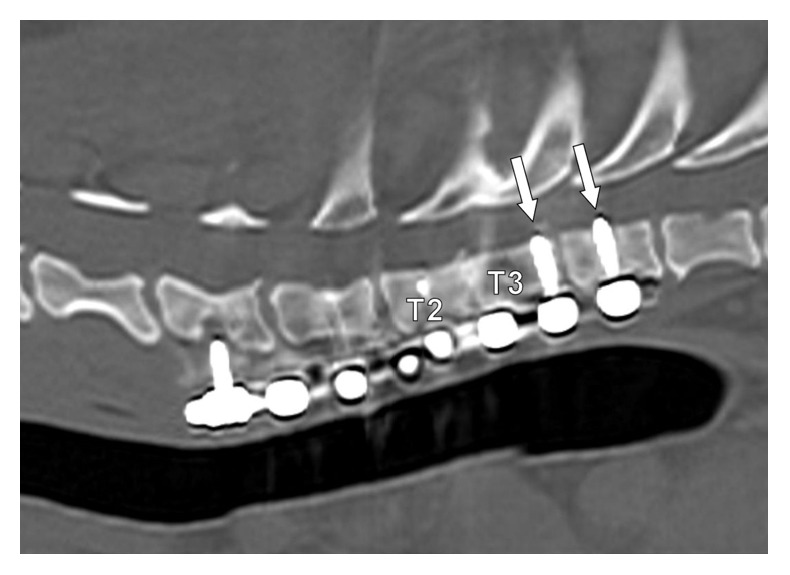
This image is a gap corrected reconstructed CT parasagittal slice of the dog's spine eight weeks postoperatively. The T2-T3 disc space remained collapsed but the degree of luxation was consistent with preoperative imaging. The two caudal screws are noted to protrude slightly into the spinal canal (white arrows).

CT of the cervical and thoracic spine was performed under sedation using dexmedetomidine (14mcg/kg IM) and reversed with atipamezole (0.25mg/kg IM). The intervertebral disc of T2-T3 remained collapsed, the luxation of T2-T3 was consistent with initial postoperative radiographs, and the T2 caudal endplate fracture was ill-defined. The two most distal screws protruded into the vertebral canal but spinal cord compression was not suspected (Figure 7).

## 3. Discussion

In this case, the dog's lesion was neurolocalized to T3-L3 with suspected C6-T2 involvement with advanced imaging isolating the lesion to T2-T3. The neurologic deficits of absent pelvic limb proprioception, pelvic limb paraplegia, and panniculus cut-off at L1 with maintenance of reflexes in all limbs suggest a neurolocalization of T3-L3; though, lateral recumbency, thoracic limb paresis, and absent proprioception indicate a cervical lesion [15]. The lesion in this case is in an unusual location as it does not fit into a traditionally accepted category of neurolocalization; rather it falls between the C6-T2 and T3-L3 regions. The thoracic limb signs and lateral recumbency were likely due to a combination of involvement of the spinal segments feeding the radial nerve and interruption of ascending inhibition causing Schiff-Sherrington rigidity. Additionally, the radial nerve carries most of the proprioceptive information from the thoracic limb, explaining the proprioceptive deficits.

As previously mentioned, the T2-T3 luxation and associated endplate fracture in this dog represent an unusual traumatic injury. The cranial thoracic column has an inherent rigidity, making vertebral fractures and luxations uncommon, minor, and often amenable to medical management [3, 6]. The attachment of ribs, intercostal and epaxial musculature, and ligamentous support all contribute to this stability [6].

A variety of stabilization techniques have been described along the spine including vertebral body plating (dorsolateral in the lumbar and ventral in the cervical regions), vertebral stapling, modified segmental fixation, cross pinning, external skeletal fixation (ESF), and a composite of PMMA and pins or screws [1, 3–13]. While all of these methods are an effective means of spinal fixation, they have limited applicability in the cranial thoracic region. Further, internal fixation of cranial thoracic injuries is not typically warranted and thus methods are poorly described [3].

Surgery of the cranial thoracic vertebrae is challenging. Adequate exposure of vertebral bodies cranial to T12 is difficult to achieve through the traditional dorsal approach [5, 13]. Dissection must be deep to the epaxial musculature to the level of the costovertebral junction, greatly limiting the visibility and angles of approach [4, 11]. There is also a high risk of iatrogenic trauma to the underlying structures including spinal nerve roots and vessels, great vessels, and inducing an iatrogenic pneumothorax [4, 5, 8, 9, 11, 13].

Application of traditional methods was considered but ultimately declined in this case. Placement of a plate on the dorsolateral aspect of a vertebral body has been shown to be a highly effective solution for luxations, subluxations, and vertebral fractures in the lumbosacral region, while a ventral approach of the cervical region is equally effective [5–7, 12, 13]. However, dorsolateral plate application in the cranial thorax is anatomically challenging due to reasons previously mentioned, as well as the requirement for rib head resection at the costovertebral junction [5, 7, 13]. Size constraints and configuration are likewise challenging, as holes must be lined up perfectly to adequately engage cortex while avoiding inadvertent tissue damage [6]. While using a dorsolateral plate in this dog was feasible, it was not ideal.

The use of PMMA composites has gained traction in spinal surgery as it overcomes limitations seen with vertebral body plates. It does not require rib head resection and few limitations of pin size, length, or placement exist [4–7]. While dorsal spinal stabilization with pins has been reportedly successful when placed at angles ranging from 20 to 45 degrees from midline, the depth of the surgical field necessary to access T2-T3 would greatly limit the angle of insertion achievable, likely under 20 degrees, thus raising concerns of increasing the spinal instability in this dog [7]. Intrathoracic placement of pins and PMMA has been described for mid thoracic spinal trauma, but the trajectory of the pins in the thoracic cavity, close proximity of vessels during the placement, and exothermic curing of PMMA raised concerns [16].

ESF is an effective option for providing alignment and stability for both vertebral luxation and fracture. While there is risk of inadvertent trauma to underlying structures, ESF does not require complete exposure of vertebrae for pin placement. This decrease of tissue dissection preserves both muscle bodies and attachment sites, maintaining the inherent stability of the thoracic column. Additionally, pins can be placed further from the injury site and the structure is removable once the spine has healed. In previous reports, ESF has solely been described in the lumbar region, with a 45-degree angle from midline being the smallest reported degree of insertion [8–10]. As with PMMA, the angle of pin placement is limited in the cranial thorax, raising the question of stability of such a structure for this dog. Additionally, the relatively long distance from the connecting bar to the vertebral bodies for a thoracic stabilization such as the one in this report would negatively affect the stiffness of the construct.

These traditional methods all utilize a dorsal approach to the spine which has inherent risks to the underlying structures. Alternative approaches have been described with reports of stabilizing vertebral fractures of T5 and T11 through a median sternotomy and lateral intercostal approach, respectively [16, 17].

A ventral approach with placement of a plate was chosen for this dog. Based on structure alone, the ventral aspects of thoracic vertebral bodies provide a flatter surface than the traditionally used dorsolateral region, and both the costochondral junction and vertebral processes are no longer a concern. An SOP plate was selected for its advantages over traditional plates, including allowing 6 degrees of freedom in contouring thus facilitating use on irregularly shaped bones while maintaining strength, and not relying on plate-bone friction for strength of the construct [11, 12, 16]. Lastly the design of the SOP facilitates close side by side juxtapositioning of plates, allowing double plating in a narrow region. Unlike SOP, traditional plates have not been found to offer enough versatility to appropriately fit thoracic vertebrae [12].

The endplate fracture fragment within the spinal canal was not addressed as removal remains controversial and is currently thought to be unnecessary [3].

## 4. Conclusion

This report demonstrates the successful use of ventral vertebral body plating via a median sternotomy. This approach allowed for satisfactory exposure of the vertebral bodies both cranial and caudal to the site of injury as well as safe application of the implants. Vital intrathoracic structures posed a potential risk for the use of power tools; however these structures were adequately retracted and protected from iatrogenic injury. After stabilization, the dog in this report made a satisfactory functional recovery, though neurologic deficits persisted. Though this type of injury may be infrequent, this approach and stabilization technique warrants consideration.

## Data Availability

No data were used to support this case report.

## Abbreviations

PMMA: Polymethylmethacrylate
MRI: Magnetic resonance imaging
T2-WI: T2 weighted image
STIR: Short tau inversion recovery
CT: Computed tomography
SOP: String-of-Pearls
ESF: External skeletal fixation.
